# A general species delimitation method with applications to phylogenetic placements

**DOI:** 10.1093/bioinformatics/btt499

**Published:** 2013-08-29

**Authors:** Jiajie Zhang, Paschalia Kapli, Pavlos Pavlidis, Alexandros Stamatakis

**Affiliations:** ^1^The Exelixis Lab, Scientific Computing Group, Heidelberg Institute for Theoretical Studies, D-68159 Heidelberg, Germany, ^2^Graduate School for Computing in Medicine and Life Sciences, University of Lübeck, ^3^Institut für Neuro- und Bioinformatik, University of Lübeck, 23538 Lübeck, Germany, ^4^Natural History Museum of Crete, University of Crete, GR-71409 Irakleio, Crete, Greece and ^5^Institute of Molecular Biology and Biotechnology, Foundation for Research and Technology-Hellas-FORTH, GR-70013 Heraklion, Crete, Greece

## Abstract

**Motivation:** Sequence-based methods to delimit species are central to DNA taxonomy, microbial community surveys and DNA metabarcoding studies. Current approaches either rely on simple sequence similarity thresholds (OTU-picking) or on complex and compute-intensive evolutionary models. The OTU-picking methods scale well on large datasets, but the results are highly sensitive to the similarity threshold. Coalescent-based species delimitation approaches often rely on Bayesian statistics and Markov Chain Monte Carlo sampling, and can therefore only be applied to small datasets.

**Results:** We introduce the Poisson tree processes (PTP) model to infer putative species boundaries on a given phylogenetic input tree. We also integrate PTP with our evolutionary placement algorithm (EPA-PTP) to count the number of species in phylogenetic placements. We compare our approaches with popular OTU-picking methods and the General Mixed Yule Coalescent (GMYC) model. For *de novo* species delimitation, the stand-alone PTP model generally outperforms GYMC as well as OTU-picking methods when evolutionary distances between species are small. PTP neither requires an ultrametric input tree nor a sequence similarity threshold as input. In the open reference species delimitation approach, EPA-PTP yields more accurate results than *de novo* species delimitation methods. Finally, EPA-PTP scales on large datasets because it relies on the parallel implementations of the EPA and RAxML, thereby allowing to delimit species in high-throughput sequencing data.

**Availability and implementation:** The code is freely available at www.exelixis-lab.org/software.html.

**Contact:** Alexandros.Stamatakis@h-its.org

**Supplementary information:** Supplementary data are available at *Bioinformatics* online.

## 1 INTRODUCTION

DNA barcoding studies mostly rely on a single marker gene and are widely used for *DNA taxonomy* (Goldstein and DeSalle, 2011; Vogler and Monaghan, 2007). More recently, high-throughput sequencing of barcoding genes has been deployed to disentangle the structure of microbial communities (Caporaso *et al.*, 2011) and in *metabarcoding* biodiversity (Coissac *et al.*, 2012) studies. A central analytical task in such studies is to classify molecular sequences into entities that correspond to species; this is commonly denoted as OTU-picking in metagenomic studies (Sun *et al.*, 2012). The main goals of such methods are to identify known species and delimit new species (Vogler and Monaghan, 2007).

Numerous approaches exist for associating anonymous reads/query sequences with known species, for instance, nearest-neighbor BLAST (Liu *et al.*, 2008) or the naïve Bayesian classifier (Wang *et al.*, 2007). These methods use sequence similarity to associate reads with taxonomic ranks. Phylogeny-aware methods for identifying reads were introduced independently and simultaneously with the evolutionary placement algorithm (EPA; Berger *et al.*, 2011) and pplacer (Matsen *et al.*, 2010). Instead of sequence similarity, they use the phylogenetic signal in the reference *and* query sequences to attain higher classification accuracy. Note that obtaining a taxonomic classification from phylogenetic placements represents a difficult task because phylogenies and taxonomies are frequently incongruent (Cole *et al.*, 2009). Placement methods are similar to *closed-reference* OTU-picking (Bik *et al.*, 2012) or *taxonomy-dependent* methods (Schloss and Westcott, 2011). Their ability to associate query sequences with species depends on the completeness of the taxon sampling in the reference data (Meyer and Paulay, 2005). Closed-reference or taxonomy-dependent methods generally lack the ability to delimit new species; consequently, they may underestimate the number of species and hence the diversity in the query sequences (see example in Supplementary Fig. S1).

To identify new species, *taxonomy-independent* methods or *de novo* OTU-picking approaches are used to initially cluster sequences into so-called molecular operational taxonomic units (MOTUs) (Floyd *et al.*, 2002; Goldstein and DeSalle, 2011; Vogler and Monaghan, 2007). Then, one can use a representative sequence from each MOTU cluster and assign a taxonomic rank via taxonomy-dependent methods. Although taxonomic assignments may still be inaccurate due to incomplete reference data, coarse-grain biodiversity estimates can be accurate when MOTUs are assigned to higher taxonomic ranks. *De novo* OTU-picking usually relies on unsupervised machine learning methods (Cai and Sun, 2011; Edgar, 2010; Fu *et al.*, 2012) that cluster sequences based on, mostly arbitrary, sequence similarity thresholds (Puillandre *et al.*, 2012; Schloss and Westcott, 2011). However, it is currently unclear how MOTUs correspond to species (Vogler and Monaghan, 2007).

To delimit species using molecular sequences, we initially need to define our species concept. The phylogenetic species concept (PSC) was initially introduced by Eldredge and Cracraft (1980) and subsequently refined by Baum and Donoghue (1995); Cracraft (1983); Davis and Nixon (1992); and Nixon and Wheeler (1990). For a review of PSCs definitions please refer to Baum and Shaw (1995). In general, phylogenetic species are the smallest units for which phylogenetic relationships can be reliably inferred. The PSC, in particular, from the genealogical point of view (Baum and Shaw, 1995), states that species reside at the transition point between evolutionary relationships that are best represented phylogenetically and relationships that are best reflected by reticulating genealogical connections (Goldstein and Desalle, 2000).

There already exist several PSC-based species delimitation approaches (e.g. see reviews in Fujita *et al.*, 2012; Sites and Marshall, 2003, 2004). The General Mixed Yule Coalescent (GMYC) model (Fujisawa and Barraclough, 2013; Pons *et al.*, 2006) for delimiting species on single genes is frequently used in empirical studies (Carstens and Dewey, 2010; Fontaneto *et al.*, 2007; Monaghan *et al.*, 2009; Powell, 2012; Vuataz *et al.*, 2011).

The GMYC method models speciation (among-species branching events) via a pure birth process and within-species branching events as neutral coalescent processes. GMYC identifies the transition points between inter- and intra-species branching rates on a time-calibrated ultrametric tree by maximizing the likelihood score of the model. It assumes that all lineages leading from the root to the transition points are different species. GMYC has been shown to work well for comparatively small population sizes and low birth rates (Esselstyn *et al.*, 2012). One drawback of GMYC is that it depends on the accuracy of the ultrametric input tree. Obtaining an ultrametric tree from a given phylogeny is a compute-intensive *and* potentially error-prone process. Most state-of-the-art likelihood-based tree calibration methods such as BEAST (Drummond and Rambaut, 2007) or DPPDIV (Heath *et al.*, 2012) rely on Bayesian sampling using MCMC (Markov Chain Monte Carlo) methods. When delimiting species in phylogenetic placements, which requires calibrating thousands of trees, it is almost impossible to deploy these methods in an automated pipeline, given the difficulties to assess MCMC chain convergence, for instance.

Inspired by the PSC, we introduce the PTP model that can delimit species using non-ultrametric phylogenies. Ultrametricity is not required because we model speciation rate by directly using the number of substitutions. The PSC implies that deploying phylogenetic reconstruction methods within a species is inappropriate. A hierarchical relationship can nonetheless be inferred for intra-species sequences using phylogenetic methods. However, we expect to observe significant (in the statistical sense) differences between the relationships reconstructed among and within species. These differences are reflected by branch lengths that represent the mean expected number of substitutions per site between two branching events. Thus, our fundamental assumption is that the number of substitutions between species is significantly higher than the number of substitutions within species. In a sense, this is analogous to the GMYC approach that intends to identify significant changes in the pace of branching events on the tree. However, GMYC uses time to identify branching rate transition points, whereas, in contrast, PTP directly uses the number of substitutions.

PTP is simple, fast and robust. Thus, it can easily be integrated with the EPA to calculate the number of species in a set of query sequences that have been placed into a specific branch of the reference phylogeny. We implemented an open reference species delimitation pipeline by integrating PTP with the EPA to identify known and new species.

Initially, we assess the performance of GMYC and the PTP approach as general *de novo* species delimitation methods using real and simulated data. We then compare PTP and GMYC with two representative OTU-picking methods UCLUST (Edgar, 2010) and CROP (Hao *et al.*, 2011). UCLUST represents a fixed threshold OTU-picking approach, whereas CROP is a soft threshold method that attempts to detect sequence clusters using a Gaussian mixture model. Finally, we evaluate the performance of our open reference approach EPA-PTP. For a fair comparison, we also integrated CROP with the EPA (EPA-CROP). 

## 2 METHODS

### 2.1 The Poisson tree processes model

Classic speciation models such as the birth–death process (BDP) assume that new species will emerge and current species will become extinct at certain rates that are measured in unit time (Barraclough and Nee, 2001). Usually, a time-calibrated tree is required as an input. Thus, for molecular sequence data, a molecular clock model must be applied to calibrate the tree. Coalescent theory also relies on unit time to describe the relationships among ancestors and descendants in a population.

Instead, we may consider the number of substitutions between branching and/or speciation events, by modeling speciations using the number of substitutions instead of the time. The underlying assumption is that each substitution has a small probability of generating a speciation. Note that the substitutions are independent of each other. If we consider one substitution at a time in discrete steps, the probability of observing η speciations for κ substitutions is given by a binomial distribution. Because we assume that each substitution has a small probability ρ of generating a speciation, and the number of substitutions in a population of size η is large, the process follows a Poisson distribution in continuous time with rate 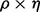. Therefore, the number of substitutions until the next speciation event follows an exponential distribution.

Comparing this with the assumptions of a BDP, we observe that each generation (e.g. with a generation time of 20 years) on a time-calibrated ultrametric tree has a small probability of speciation. The BDP does not model substitutions, thus, substitutions are superimposed onto the BDP, whereas PTP explicitly models substitutions. Substitution information can easily be obtained by using the branch lengths of the phylogenetic input tree. Thus, in our model, the underlying assumptions for observing a branching event are consistent with the assumptions made for phylogenetic tree inference.

We can now consider two independent classes of Poisson processes. One process class describes speciation such that the average number of substitutions until the next speciation event follows an exponential distribution. Given the species tree, we can estimate the rate of speciations per substitution in a straightforward way. The second Poisson process class describes within-species branching events that are analogous to coalescent events. We assume that the number of substitutions until the next within-species branching event also follows an exponential distribution. Thus, our model assumes that the branch lengths of the input tree have been generated by two independent Poisson process classes.

**Fig. 1.**
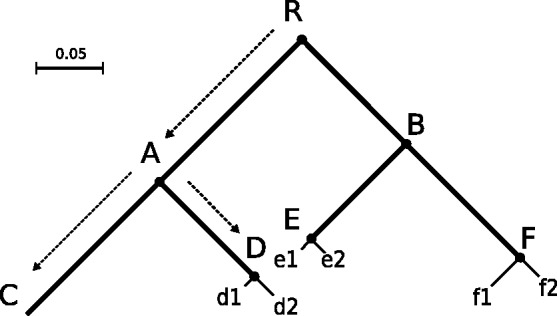
Illustration of the *PTP*. The example tree contains 6 speciation events: *R, A, B, D, E, F*, and 4 species: *C, D, E, F*. Species *C* consists of one individual; species *D, E, F* have two individuals each. The thick lines represent among-species *PTP*, and the thin lines represent within-species *PTPs*. The Newick representation of this tree is ((*C:0.14, (d1:0.01, d2:0.02)D:0.1)A:0.15, ((e1:0.015, e2:0.014)E:0.1, (f1:0.03, f2:0.02)F:0.12)B:0.11)R*. The tree has a total of 16 different possible species delimitations. The maximum likelihood search returned the depicted species delimitation with a log-likelihood score of 24.77, and 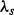 = 8.33 and 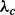 = 55.05

In the following step, we assign/fit the Poisson processes to the tree. Let *T* be a rooted tree, and *P_i_* be a path from the root to leaf *i*, where 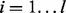 and *l* is the number of leaves. Let 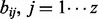 be the edge lengths of *P_i_*, representing the number of substitutions. We further assume that 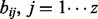 are independent exponentially distributed random variables with parameter λ. Let 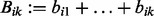 be the sum over the edge lengths for 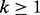. We further define 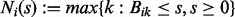. *B_ik_* is the number of substitutions of the *k*th branching event, and 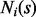 is the number of branching events below *B_ik_*. Note that 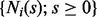 constitutes a Poisson process. Thereby, *T* and 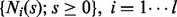 together form a tree of Poisson processes, which we denote as *Poisson Tree Processes (PTP)*. To a rooted phylogeny with *m* species, we apply/fit one among-species PTP and at most *m* within-species PTPs. An example is shown in Figure 1.

In analogy to BP&P (Yang and Rannala, 2010) and GMYC (Pons *et al.*, 2006), we conduct a search for the transition points where the branching pattern changes from an among-species to a within-species branching pattern. The total number of possible delimitations on a rooted binary tree with *m* tips ranges between *m* (caterpillar tree) and 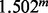, depending on the actual tree shape (Fujisawa and Barraclough, 2013). Because the search space is generally too large for an exhaustive search, we need to devise heuristic search strategies. Given a fixed species delimitation, we fit two exponential distributions to the respective two branch length classes (among- and within-species branching events). We calculate the log-likelihood as follows:
(1)
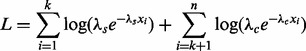

where *x*_1_ to *x_k_* are the branch lengths generated by among-species PTPs, 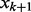 to *x_n_* are the branch lengths of within-species PTPs, 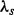 is the speciation rate per substitution and 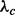 is the rate of within-species branching events per substitution. The rates 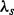 and 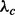 can be obtained via the inverse of the average branch lengths that belong to the respective processes. Based on Equation (1), we search for the species delimitation that maximizes *L*. A standard likelihood-ratio test with one degree of freedom can be used to test if there are indeed two classes of branch lengths. Large *P*-value indicates that either all sequences are one species or that every sequence represents a single species.

We developed and assessed three heuristic search strategies for finding species delimitations with ‘good’ likelihood scores, which are described in the online supplement. For the experimental results presented here, we used the heuristic that performed best, based on our preliminary experiments.

### 2.2 Species delimitation using phylogenetic placements

In the following, we describe the open reference species delimitation pipeline that combines the EPA with the PTP (EPA-PTP). The EPA initially places a large number of query sequences (short reads) into the branches of a given reference phylogeny. Thereafter, we execute PTP separately and independently for the query sequences assigned to each branch. This allows to annotate the branches of the reference tree by the number of species induced by the query sequences that were placed into each branch. The input of our pipeline is a reference alignment where each sequence represents one species and a reference phylogeny for that alignment. The PTP method and the pipeline are implemented in Python and rely on the python Environment for Tree Exploration package (Huerta-Cepas *et al.*, 2010) for tree manipulation and visualization.

Our pipeline executes the following steps:

1. Run UCHIME (Edgar *et al.*, 2011) against the reference alignment to remove chimeric query sequences.
2. Use EPA to place the query sequences onto the reference tree. Sequences that have a maximum placement likelihood weight of <0.5 (i.e. an uncertain placement, see Berger *et al.*, 2011 for details) are discarded.
3. For each branch in the reference tree, we extract the set of query sequences that have been placed into that branch and infer a tree on them using RAxML (Stamatakis, 2006). Because the PTP method requires a correctly rooted tree, we use the following two rooting strategies: if the branch leads to a tip, apart from the query sequences, we extend the alignment by including the reference tree tip sequence and that reference sequence that is furthest away from the current tip. The most distant sequence is used as outgroup. Keep in mind, that thereby the tree will be rooted at the longest branch (see the discussion below). To analyze query sequence placements at internal branches, we use the RAxML −g constraint tree option to obtain a rooted tree of the query sequences. The constraint tree consists of the bifurcating reference tree and a polytomy comprising the query sequences attached to the reference tree branch under consideration. The result of this constrained ML tree search is a resolved tree of query sequences that are attached to the reference tree branch. The attachment point is used as root.
4. Because we assume that the reference phylogeny is a species tree that reflects our knowledge about the speciation process and rate, we initially estimate 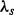 only once on the reference phylogeny. Thereafter, we apply PTP to each query sequence (one for each branch of the reference phylogeny) tree to delimit species. Note that in this scenario we will only need to estimate 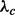, as 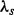 remains fixed.
5. When PTP is applied to a placement of query sequences on a terminal branch, those queries that are delimited as one population with the reference sequence at the tip will be assigned taxonomically to the species represented by this reference sequence. Otherwise, they are identified as new species in the reference tree.

As mentioned previously, we also combined EPA with CROP (EPA-CROP). The method works as EPA-PTP, with the only difference that CROP is used instead of PTP to calculate the number of MOTUs for each placement. 

## 3 EXPERIMENTAL SETUP

We initially tested stand-alone PTP for general species delimitation and compared it with the single-threshold GMYC model. The single-threshold GMYC model infers a single cutoff time *T* where all nodes above *T* represent species. Although the more advanced multiple-threshold GMYC allows for several threshold times *T_i_*, the single-threshold GMYC is usually more accurate than the multi-threshold version (see Fujita *et al.*, 2012 for details). For simulated data, we used RAxML (Stamatakis, 2006) to infer phylogenetic trees, and then used them as input to PTP. Subsequently, these phylogenies were made *ultrametric* by r8s (Sanderson, 2003) to test GMYC. For UCLUST and CROP, only molecular sequences are needed as input. In both programs, we set the sequence dissimilarity threshold to 97%, a widely accepted threshold for bacterial sequences (Stackebrandt and Goebel, 1994). For real datasets, we used the phylogenetic tree and ultrametric tree from the original publications whenever possible, otherwise we used the same settings as described previously.

We then tested our open reference species delimitation approaches (EPA-PTP and EPA-CROP). We also assessed the impact of incomplete taxon sampling on the accuracy of these approaches, by removing up to 50% of the reference sequences.

### 3.1 Empirical datasets

#### 3.1.1 Arthropod datasets

The *Rivancidella* dataset comprises three genes (cyt b, COI, 16S) and was originally used in Pons *et al.* (2006). The total number of sequences is 472, which represents 24 morphological species and 4 outgroup taxa. The estimated number of putative species for the genus as inferred by GMYC was 48 (with confidence limits ranging between 46 and 52 species). Alternative methods (see Pons *et al.*, 2006 for details) used in this study yielded 46 and 47 putative species, respectively.

We also used COI marker datasets (Papadopoulou *et al.*, 2010) of the genera *Dendarus*, *Pimellia* and *Tentyria*. The datasets comprise 51, 56 and 59 sequences, respectively. The number of species that were attributed to each taxon using morphological criteria was seven, one and one.

#### 3.1.2 Gallotia dataset

The lizard genus *Gallotia* comprises seven species (based on genetic *and* morphological markers) that are endemic to the Canary islands. The taxonomic species tree and the molecular phylogeny for this dataset are fully congruent. The data (Cox *et al.*, 2010) comprises four mitochondrial genes (cyt b, COI, 12S, 16S) and a total of 90 sequences (76 representing *Gallotia* and 14 outgroup sequences). 

#### 3.1.3 Arthropod metabarcoding dataset

This dataset contains 673 full-length COI arthropod sequences with a length of 658 bp. The sequence was obtained via polymerase chain reaction amplification and Sanger sequencing. Subsequently, these 673 sequences were resequenced with a 454 sequencer to generate a total of 133 057 short reads (Yu *et al.*, 2012). Using the Sanger data as reference, Yu *et al.* developed metabarcoding protocols that use the 454 reads to unravel the diversity in the reference data. The authors use a multistep OTU-picking procedure with different similarity thresholds for clustering the 454 reads and the full-length reference sequences. The method clustered the 673 sequences into 547 MOTUs. The OTU-picking results for the 454 data are summarized in Table 4. Our PTP model finds 545 putative species in the 673 full-length sequences when directly applied to the phylogenetic reference tree. To ensure comparability of results, we used the 547 MOTUs identified in the original study to build a reference tree and reference alignment for testing the EPA-PTP and EPA-CROP pipelines. Initially, we aligned 454 sequences with a length exceeding 100 bp to these 547 reference sequences with HMMER (Eddy, 2009). Yu *et al.* initially blasted the 454-MOTU (obtained via three alternative clustering methods) to the Sanger-MOTUs using a threshold of 1e-10 and 97% minimum similarity. The Sanger-MOTUs that did not match any of the 454-MOTUs are called ‘dropouts’ by the authors. Inversely, 454-MOTUs that did not match Sanger-MOTUs are called ‘no-matches’.

Analogously, in our pipelines, when the delimited species from 454 sequence placements contain one of the full-length reference sequences (see step 4 in 2.2), we consider this as a ‘match’. Further, we denote a full-length reference sequence that is not included in any short read placement delimitation as ‘dropout’. Finally, we call a short read placement that is delimited as a new species (i.e. does not contain a reference sequence) as ‘no-match’.

### 3.2 Simulations

We generated simulated datasets using a Yule coalescent model. We used ms (Hudson, 2002) and BioPerl (Stajich *et al.*, 2002) in combination with INDELible (Fletcher and Yang, 2009) to simulate sequences. Using a modified version of the BioPerl module Bio::Phylo that allows to vary the birth rate value in the simulations, we initially generated a set of random *species* trees 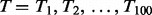. The leaves of each tree *T_i_* (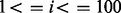) represent extant species. All 600 simulated datasets we generated contain 30 species. In the next step, we used ms to generate a structured coalescent gene tree. The node ages of the phylogenetic tree *T_i_* are interpreted as divergence times between populations. In other words, we treat species as diverged populations that were completely isolated from each other after they diverged from their common ancestor. Thus, using ms we simulated a multispecies coalescent gene tree with 30 species and 100 individuals per species. For each species, we randomly selected 10 individuals to generate evenly sampled (in terms of the number of individuals per species) datasets. We also generated unevenly sampled datasets containing 2 species with 100 individuals, 4 with 50 individuals, 8 with 10 individuals and 16 with 2 individuals. Finally, we used INDELible to simulate DNA alignments of 250, 500 and 1000 bp on the previously mentioned multispecies coalescent trees.

We generated datasets with a scaled birth rate (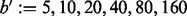); small values generate large evolutionary distances between species. For details on the simulations and on the scaled rate 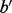, please refer to the online supplement.

We used the normalized mutual information (NMI) criterion (Vinh *et al.*, 2010) to asses how the delimitation accuracy of the different algorithms agrees with the ground truth. The mutual information (MI) of two distinct partitions of the same dataset quantifies how much information is shared by these; NMI scales MI to values between 0.0 and 1.0. In our case, NMI = 1 means that the delimitation is identical to the ground truth, whereas NMI = 0 means that the delimited species are randomly partitioned compared with the ground truth.

Finally, we also tested the EPA-PTP and EPA-CROP pipelines on simulated data. In each simulated alignment, we randomly selected one individual sequence per species as reference sequence and treated the remaining sequences (of that species) as query sequences. To assess the impact of incomplete reference trees on species delimitations, we randomly removed up to 50% of the reference sequences. We deployed the same metrics as mentioned previously to quantify delimitation accuracy.

## 4 RESULTS

### 4.1 General species delimitation

The number of putative species delimited for *Dendarus*, *Pimelia*, *Tentyria* and *Gallotia* are comparable for all four methods (Table 1). For the *Gallotia* dataset, GMYC and PTP yield identical results. Three of the *Gallotia* species were split into two separate groups according to geographical isolation of the corresponding populations on different islands (see Supplementary Fig. S4).

**Table 1.** Number of species delimitated on real data

Taxon	Morphological	GMYC	PTP	CROP	UCLUST
*Rivacindela*	24	48	27/44^a^	6	82
*Dendarus*	7	10	9/11^a^	7	11
*Pimelia*	1	10	9/15^a^	7	10
*Tentyria*	1	2	2/2^a^	1	3
*Gallotia*	7	10	10/10^a^	9	15

^a^Using the ultrametric tree as an input for PTP.

On the *Rivacindela* dataset PTP yields a more conservative delimitation than GMYC. PTP identifies 27 putative species (GMYC: 48), which is closer to the number of morphological species (24) and the number of independent networks (25) obtained via statistical parsimony (Pons *et al.*, 2006). This pronounced difference may be associated with the construction of the ultrametric tree. According to the r8s manual, the presence of many short (close to zero) branches in the tree can yield inaccurate results. When applying PTP to the ultrametric tree, the resulting estimate is substantially closer to the GMYC estimate (see Table 1). Thus, we believe that the overestimation of the *Rivacidela* species by GMYC is most probably because of an erroneous ultrametric tree reconstruction. CROP and UCLUST yield dissimilar results; CROP only detects 6 clusters, whereas UCLUST detects 82 clusters. 

The results on evenly sampled simulated data are summarized in Table 2 and Supplementary Table S1. On average, PTP shows the best performance and outperforms GMYC in all the test scenarios. OTU-picking methods work well on datasets with small 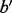 values that is when the evolutionary distances between species are large. For 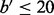, UCLUST generally outperforms PTP and yields the best overall results. However, with increasing 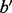 the accuracy of OTU-picking methods decreases steeply. As expected, for shorter sequence lengths (250 and 500 bp), accuracy deteriorates for all methods and in a more pronounced way for PTP and GMYC. However, even with sequence lengths of 250 bp, PTP still yields best results on datasets with 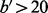.

**Table 2.** Species delimitation accuracy (measured in NMI) on simulated evenly sampled data

NMI	b′						Mean (variance)
	5	10	20	40	80	160	
1000 bp							
UCLUST	0.969	0.959	0.938	0.892	0.782	0.575	0.852 (0.023)
CROP	0.964	0.930	0.848	0.646	0.232	0.038	0.609 (0.151)
GMYC	0.924	0.914	0.907	0.886	0.834	0.697	0.860 (0.007)
PTP	0.944	0.935	0.922	0.905	0.882	0.857	0.907 (0.001)
250 bp							
UCLUST	0.967	0.954	0.930	0.871	0.735	0.522	0.829 (0.029)
CROP	0.961	0.917	0.800	0.545	0.152	0.024	0.566 (0.159)
GMYC	0.892	0.620	0.484	0.464	0.550	0.503	0.585 (0.025)
PTP	0.946	0.927	0.907	0.881	0.833	0.780	0.879 (0.003)

On the unevenly sampled simulated datasets (Supplementary Table S2), the delimitation accuracy decreases for UCLUST and PTP. CROP and GMYC yield higher NMI scores than on evenly sampled dataset. On average, PTP yields the best results over all (evenly and unevenly sampled) simulated datasets.

### 4.2 Species delimitation with phylogenetic placements

By combining EPA with PTP (or CROP) and applying it to simulated data as described in Section 3.2, we can substantially improve the delimitation accuracy on simulated data (Table 3 and Supplementary Tables S3 and S5).

**Table 3.** Species delimitation accuracy (measured in NMI) on simulated evenly sampled data using the EPA-PTP pipeline

NMI	b′						Mean (variance)
	5	10	20	40	80	160	
1000 bp							
Full ref.	0.989	0.978	0.962	0.933	0.884	0.836	0.930 (0.003)
90% ref.	0.984	0.972	0.955	0.925	0.876	0.830	0.923 (0.003)
80% ref.	0.976	0.966	0.949	0.921	0.872	0.823	0.917 (0.003)
70% ref.	0.971	0.959	0.943	0.912	0.868	0.816	0.911 (0.003)
60% ref.	0.966	0.956	0.939	0.908	0.860	0.805	0.905 (0.003)
50% ref.	0.962	0.950	0.934	0.904	0.853	0.787	0.898 (0.004)
250 bp							
Full ref.	0.978	0.968	0.949	0.918	0.863	0.811	0.914 (0.004)
90% ref.	0.967	0.955	0.935	0.907	0.854	0.800	0.903 (0.004)
80% ref.	0.956	0.944	0.926	0.895	0.846	0.786	0.892 (0.004)
70% ref.	0.942	0.926	0.912	0.880	0.830	0.773	0.877 (0.004)
60% ref.	0.927	0.911	0.893	0.861	0.813	0.755	0.860 (0.004)
50% ref.	0.909	0.891	0.871	0.838	0.784	0.732	0.837 (0.004)

*Note*: ref. indicates reference sequences

When the reference phylogeny includes >70% of the reference data, EPA-PTP outperforms all competing approaches, including stand-alone PTP. EPA-PTP outperforms PTP even when the reference phylogeny contains only 50% of the simulated reference data for 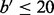. With increasing 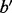, the reference data need to be more complete for EPA-PTP to outperform PTP. This is because with increasing 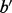, internal branch lengths tend to get shorter and the EPA placement accuracy decreases. Hence, more data are needed to obtain accurate placements. Note that under extremely high speciation rates, EPA-PTP performs worse than PTP. The estimation errors may also because of (i) discarding sequences with low likelihood weights (see Section 2.2) (ii) errors in phylogenetic inferences or (iii) PTP heuristics failing to find the maximum likelihood species delimitation.

The results for the EPA-CROP pipeline are shown in Supplementary Tables S4 and S6. EPA-CROP outperforms the stand-alone version of CROP, but the results are worse than for EPA-PTP. 

On the *Arthropod* metabarcoding data, the EPA-PTP pipeline yields substantially better results than the multistep OTU-picking pipeline used in the original publication (Table 4). When the complete full-length reference sequence tree is used, the EPA-PTP pipeline shows substantially lower ‘dropout’ and ‘no-match’ rates. It recovers 12.5% more species with respect to the reference data that represent an improvement of over 50%. Here, we apply an analogous criterion as in the original study where at least two reads need to be contained in an OTU cluster for it to be considered. In our case, 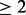 reads need to be contained in a species delimitation. If an OTU cluster or species delimitation only contains one read, it is highly likely that it represents a sequencing error. However, the availability of the complete reference dataset is not granted for most metabarcoding analyses. Thus, as for the simulated data, we randomly removed up to 50% of the reference sequences and reran our pipelines. We then calculated the ratios between the number of species estimated on the reduced reference data relative to the number of species estimated on the complete reference data. The results are shown in Supplementary Figure S5 in the online supplement. When species are delimited with taxonomy-dependent approaches, such as the EPA, the number of estimated species is expected to decrease with the number of species in the reference data. When combined with PTP (using 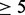 reads per delimitation as cutoff), EPA-PTP yields stable diversity estimates, irrespective of the completeness of the reference phylogeny. EPA-CROP also yields better results than the multistep OTU-picking pipeline and stand-alone CROP. The results are slightly worse than for EPA-PTP (Supplementary Table S7).

**Table 4.** *Arthropod* dataset: number of estimated MOTUs and species for the complete reference data and tree

No. reads	OTU-picking			EPA-PTP		
	Number of cluster	Drop-out (%)	No-match (%)	Number of cluster	Drop-out (%)	No-match (%)
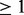 reads	973	19	42.8	587	7.3	13.6
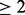 reads	602	24	25.4	516	11.5	6.2
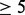 reads	—	36	—	441	21.9	3.2

*Note*: Sanger data (the reference dataset) has a total of 547 MOTUs. The ‘—’ indicates that the number is not available in the original publication.

## 5 DISCUSSION

We introduced, implemented and made available a new model (PTP) for species delimitation that is mainly intended for delimiting species in single-locus molecular phylogenies. PTP can propose species boundaries that are consistent with the PSC. An important advantage of our method is that it models speciation in terms of the number of substitutions. Thereby, it circumvents the potentially error-prone and compute-intensive process of generating time-calibrated ultrametric trees, which are required as an input for GMYC.

Using real datasets, we show that delimitations inferred with PTP are comparable with delimitations obtained via GMYC. Simulations suggest PTP outperforms GMYC.

In addition, it is more straightforward to use because it only requires a standard phylogenetic tree as input and because it also is substantially faster. On the 673-taxon metabarcoding dataset (using a modern Intel desktop processor), for instance, r8s requires 3 days to complete, whereas RAxML in combination with PTP only requires a total of about 20 min to return a species delimitation.

We also compared GMYC and PTP with two clustering algorithms: CROP and UCLUST. From our point of view, the problem of species delimitation needs to incoporate data from various sources (e.g. sequences *and* trees) and also depends heavily on the species definition used. Thus, GMYC and PTP yield comparable results on real data because they are based on the PSC. In contrast, by their very definition, CROP and UCLUST simply identify sequence clusters. The fact that there *is* a difference between sequence clusters and PSC-based species delimitation is underpinned by our simulations.

We simulate the data in accordance with the GMYC model that essentially adopts the PSC. To demonstrate the impact of the 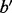 parameter on clustering-based delimitation accuracy, we plotted the pairwise sequence distances within species and between directly adjacent species in the simulated tree, for 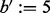 and 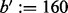 in Supplementary Figures S2 and S3 of the online supplement. Lower 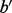 values lead to larger evolutionary distances between species, that is, the so-called barcoding gap (Puillandre *et al.*, 2012) is present. Increasing 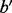 reduces the evolutionary distances between species and the barcoding gap disappears (see Puillandre *et al.*, 2012 for examples of this phenomenon on real data). Therefore, our simulations show that clustering algorithms work on datasets with the barcoding gap because phylogenetic species are mostly consistent with sequence clusters in this case. However, clustering methods are prone to fail when the barcoding gap is not present because sequences cannot be told apart any more via sequence similarity alone. As we show, GMYC and PTP delimitation performance is more robust to the absence of barcoding gap. Thus, when no prior information (barcoding gap presence) about the dataset is available and the goal is to delimit phylogenetic species, GMYC and PTP should be preferred.

Apart from the stand-alone PTP code, we also introduced the EPA-PTP pipeline that combines the EPA with PTP.

The EPA-PTP pipeline represents the first integrated approach for analyzing metagenomic data that combines the phylogenetic placement approach with an explicit statistical criterion for species delimitation. On a representative empirical dataset, our pipeline yields a substantially more accurate diversity estimate than traditional OTU-picking methods. Using simulated data, we show that, open reference-based approaches can improve delimitation accuracy compared with *de novo* approaches. More importantly, the EPA-PTP pipeline allows for deploying a widely accepted species concept to metagenomic data, where millions of sequences need to be processed. EPA-CROP (with the default setting of 2000 MCMC generations) is approximately twice as fast as EPA-PTP on the metabarcoding dataset. Note that 2000 generations may not be sufficient, and that CROP does not offer a built-in MCMC convergence assessment criterion. 

In the following, we discuss the current limitations of our approach.

Readers should keep in mind that entities delimited by PTP are putative species only. The phylogenetic trees inferred on single-gene molecular sequences are gene trees rather than species trees, albeit the hierarchical relationships above the species boundaries are expected to be mostly consistent with the species tree. However, the boundaries inferred by PTP are only approximate. Additional data need to be integrated to further validate the delimitations, such as morphological characters and multi-gene sequence data (Ence and Carstens, 2011) within an integrative taxonomy framework (Padial *et al.*, 2010; Sauer and Hausdorf, 2012). The putative species delimited by PTP, can, for instance, be used as initial hypothesis that can be further scrutinized with multilocus coalescent-based methods such as BP&P (Yang and Rannala, 2010). BP&P requires prior knowledge of species boundaries, and it represents a validation method, rather than a delimitation method. Owing to its computational complexity, BP&P can currently only handle up to 20 species. 

Compared with OTU-picking methods, PTP and EPA-PTP require substantially more CPU time because of the phylogenetic calculations. Although most OTU-picking methods can run on an off-the-shelf desktop computer, the EPA-PTP pipeline requires a multicore server for analyzing large metagenomic datasets. 

Because PTP initiates the search for the maximum likelihood delimitation at the root of the input phylogeny, the tree has to be correctly rooted to obtain accurate estimates. Also, PTP should be used with caution on datasets where the number of individuals sampled per species is unbalanced and where the over-sampled species exhibit small within-species variation (see Supplementary Tables S1 through 4).

In such cases, the inferred phylogeny will comprise both, subtrees (comprising one species and many individuals) with a large number of extremely short branches, and subtrees (comprising one species but only few individuals) with short, but not extremely short branches. Such unbalanced samples may require the introduction of a third λ parameter class of branches to accommodate (i) over-sampled within-species branches, (ii) within-species branches and (iii) among-species branches. Otherwise, the species that are not over-sampled cannot be delimited properly, that is, each individual is likely to be identified as a separate species. Hence, we either need a criterion for removing over-sampled sequences, or a criterion to decide when and how many additional classes of *PTP* (λ parameters) need to be introduced.

However, a major drawback of introducing additional *PTP* classes is that the delimitation search space becomes significantly larger. Hence, finding the maximum likelihood delimitation or a best known delimitation represents a challenging task. Thus, before extending the number of classes, we feel that more work on the design and performance of heuristic search strategies for species delimitation is required to better characterize and understand the problem. This also applies to the heuristics used in GMYC, given that the underlying optimization problems are similar.

## Supplementary Material

Supplementary Data
