# Supplementary material for: A general species delimitation method with applications to phylogenetic placements
Source: Bioinformatics. 2013 Aug 29;29(22):2869–76. doi: 10.1093/bioinformatics/btt499 (PMC3810850; doi:10.1093/bioinformatics/btt499)
Supplement: Supplementary Data [file supp_29_22_2869__index.html]

A General Species Delimitation Method with Applications to Phylogenetic Placements — A general species delimitation method with applications to phylogenetic placements — A general species delimitation method with applications to phylogenetic placements — Supplementary Data 

# A general species delimitation method with applications to phylogenetic placements

## Supplementary Data

files

**Files in this Data Supplement:**

- Supplementary Data - pdf file
