# Supplementary material for: A general species delimitation method with applications to phylogenetic placements
Source: Bioinformatics. 2013 Aug 29;29(22):2869–76. doi: 10.1093/bioinformatics/btt499 (PMC3810850; doi:10.1093/bioinformatics/btt499)
Supplement: Supplementary Data [file supp_btt499_supporting.pdf]

A General Species Delimitation Method with Applications to Phylogenetic Placements: supplementary information

J. Zhang, P. Kapli, P. Pavlidis and A. Stamatakis*

1 UNDERESTIMATION OF THE NUMBER OF SPECIES WHEN USING THE STAND-ALONE EPA

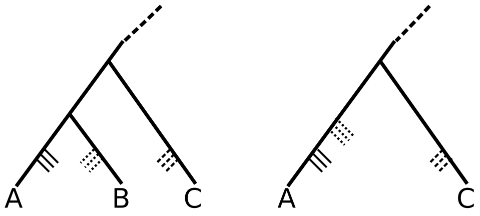

Fig. 1. EPA may underestimate the number of species in an incomplete reference phylogeny. *A*, *B*, and *C* are closely-related species. If they are all present in the reference tree, the EPA will place the corresponding query sequences onto the three respective branches leading to *A*, *B*, and *C*. However, when *B* is missing, the EPA will place query sequences belonging to *B* into the branch leading to *A*. This will incorrectly classify the query sequences belonging to *B* and thus, underestimate the number of species.

2 HEURISTICS SEARCH ALGORITHMS

Heuristic I: We order and store the branch lengths in descending order. We start with the longest branch and add one branch at a time to build consecutive sets that contain branches of among-species branching events. To each set, we add those missing branches that are required to obtain a valid species delimitation configuration, that is, span a tree starting at the root. We then evaluate the likelihood for each extended set. This approach requires $\mathcal{O}(n)$ time, where n is the number of branches in the tree. The rationale for this approach is that longer branches are more likely to form part of speciation events, rather than within-species branching events.

Heuristic II: We implement a greedy strategy that starts from the root and includes one child node at a time as speciation event via a breadth-first tree traversal. We then apply this procedure recursively by extending the child node that has the higher log likelihood score and re-considering the other child node. This heuristic has time complexity $\mathcal{O}(n^2)$. The rationale for this approach is that it uses the tree data structure to explore a larger number of possible delimitations.

Heuristic III: This hybrid approach combines the ideas of the two previous heuristics. First we order the branches as in Heuristic I. Then, we determine best bisection of this list into a within-species branch set *C* and among-species branch set *S* with respect to the likelihood score. This approach ignores the tree structure, but returns an upper bound for the likelihood score. Thereafter, we start with the longest branch again and add one branch at a time to the set *S'* of speciation event branches. In contrast to Heuristic I, the next branch we add to the set can be any branch in the original set *S* that is connected to a branch in *S'* via the tree. When no branch in *S* is connected to a branch of *S'* via the tree, we deploy the greedy strategy of Heuristic II to select the next branch we want to add. This approach combines the speed of Heuristic I with the more exhaustive search of Heuristic II.

3 SIMULATIONS

INDELible, ms, and BioPerl use different units for representing branch lengths. INDELible uses the expected number of substitutions (the standard unit in phylogenetics), whereas ms uses the coalescent time unit of $4N$ generations where N is the effective population size. BioPerl only uses the birth rate to generate trees (small birth rates generate longer trees, large birth rates generate shorter trees). We therefore converted all branch length units to the expected number of substitutions.

In our simulations, we set $\mu := 10^{-7}$, where μ is the mutation rate per base pair, per individual, and per year. This value for μ is situated approximately in the middle of the empirical value range. For instance, human genomic DNA has a rate of 10^{-8} (Nachman and Crowell, 2000), human mitochondria have a rate of 10^{-5} (Schneider and Excoffier, 1999), and viruses have a rate that ranges between 10^{-4} and 10^{-8} (Drake *et al.*, 1998).

For the birth rate b, we used a value range around 0.5 speciation events per one million years. The value of 0.5 is realistic for several distinct types of species (Mendelson and Shaw, 2005). To convert b into units of speciations per substitution we apply $b' = \frac{b}{\mu \times 10^6}$, where b' is the scaled birth rate per substitution event. Thus, values of b' around 5 can be considered as being realistic.

With respect to coalescent units, let l be a branch length in coalescent units. For an effective population size of N and a mutation rate μ, the expected number of mutations on a branch is $\frac{l}{4N\mu}$. Thus, to convert the coalescent units into the expected number of substitutions, we need to divide the branch length by $4N\mu$. Thereby, we implicitly assume that the expected number of mutations is approximately equal to the expected number of substitutions.

The key parameters for delimiting species are the birth rate and the effective population size. High birth rates decrease the evolutionary distance between species. High effective population sizes have a similar effect. This is because the coalescent rate is inversely proportional to the effective population size. When the

© Oxford University Press 2013.

population size is sufficiently large, coalescent events can occur prior to speciation and lead to incomplete lineage sorting. Thus, the effect of the birth rate on species delimitation accuracy also depends on the effective population size. Hence, the birth rate and the effective population size are not independent from each other. Therefore, we keep the effective population size constant $N := 50,000$ and investigate the effect of varying the scaled birth rate ($b' := 5, 10, 20, 40, 80, 160$).

To automate the tests, we re-implemented and also make available the single-threshold GMYC in Python. We tested the correctness of our implementation with respect to the original R implementation using the *Arthropods* and *Gallotia* data sets.

REFERENCES

Drake, J. W., Charlesworth, B., Charlesworth, D., and Crow, J. F. (1998). Rates of spontaneous mutation. *Genetics*, **148**(4), 1667–86.

Mendelson, T. C. and Shaw, K. L. (2005). Sexual behaviour: rapid speciation in an arthropod. *Nature*, **433**(7024), 375–6.

Nachman, M. W. and Crowell, S. L. (2000). Estimate of the mutation rate per nucleotide in humans. *Genetics*, **156**(1), 297–304.

Schneider, S. and Excoffier, L. (1999). Estimation of past demographic parameters from the distribution of pairwise differences when the mutation rates vary among sites: application to human mitochondrial DNA. *Genetics*, **152**(3), 1079–89.

4 COMPLETE RESULTS

Table 1. Species delimitation accuracy on simulated, evenly sampled data

NMI	b'						Mean
1000bp	5	10	20	40	80	160	(variance)
UCLUST	0.969	0.959	0.938	0.892	0.782	0.575	0.852 (0.023)
CROP	0.964	0.930	0.848	0.646	0.232	0.038	0.609 (0.151)
GMYC	0.924	0.914	0.907	0.886	0.834	0.697	0.860 (0.007)
PTP	0.944	0.935	0.922	0.905	0.882	0.857	0.907 (0.001)
500bp	5	10	20	40	80	160	
UCLUST	0.967	0.958	0.935	0.884	0.771	0.554	0.844 (0.025)
CROP	0.964	0.927	0.836	0.613	0.187	0.027	0.592 (0.158)
GMYC	0.918	0.878	0.766	0.583	0.626	0.551	0.720 (0.024)
PTP	0.952	0.938	0.920	0.898	0.864	0.828	0.900 (0.002)
250bp	5	10	20	40	80	160	
UCLUST	0.967	0.954	0.930	0.871	0.735	0.522	0.829 (0.029)
CROP	0.961	0.917	0.800	0.545	0.152	0.024	0.566 (0.159)
GMYC	0.892	0.620	0.484	0.464	0.550	0.503	0.585 (0.025)
PTP	0.946	0.927	0.907	0.881	0.833	0.780	0.879 (0.003)

Table 2. Species delimitation accuracy on simulated, unevenly sampled data

NMI	b'						Mean
1000bp	5	10	20	40	80	160	(variance)
UCLUST	0.937	0.936	0.923	0.886	0.789	0.582	0.842 (0.019)
CROP	0.971	0.946	0.892	0.723	0.303	0.047	0.647 (0.147)
GMYC	0.937	0.894	0.849	0.834	0.791	0.725	0.838 (0.005)
PTP	0.921	0.912	0.889	0.866	0.830	0.800	0.892 (0.006)
500bp	5	10	20	40	80	160	
UCLUST	0.936	0.936	0.920	0.882	0.775	0.563	0.835 (0.021)
CROP	0.971	0.945	0.875	0.682	0.232	0.031	0.622 (0.159)
GMYC	0.941	0.901	0.870	0.792	0.658	0.610	0.795 (0.018)
PTP	0.943	0.927	0.904	0.878	0.835	0.784	0.878 (0.003)
250bp	5	10	20	40	80	160	
UCLUST	0.935	0.933	0.913	0.866	0.742	0.514	0.817 (0.027)
CROP	0.970	0.937	0.852	0.616	0.192	0.021	0.598 (0.163)
GMYC	0.925	0.867	0.814	0.732	0.586	0.523	0.741 (0.025)
PTP	0.948	0.924	0.901	0.863	0.812	0.753	0.866 (0.005)

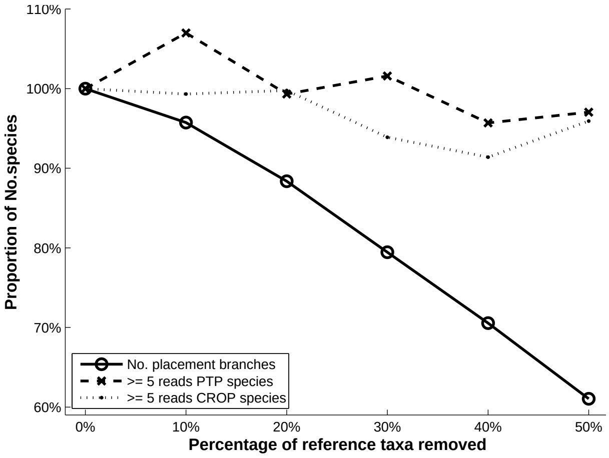

Fig. 5. Number of estimated species on incomplete reference trees on the Arthropod meta-barcoding dataset.

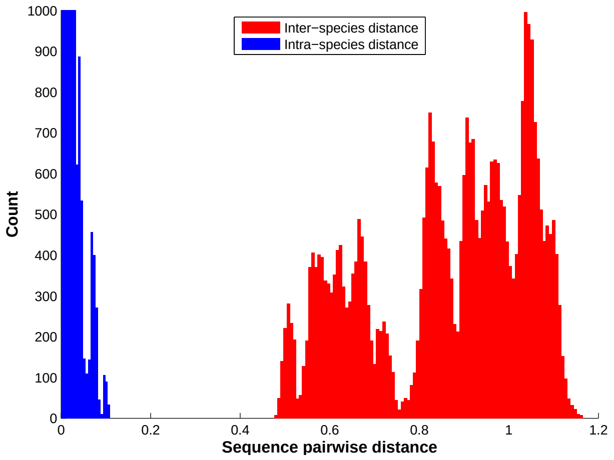

Fig. 2. Histogram of pairwise sequence distances within and among species ($b' = 5$). A clear gap exists between two types of pairwise distances, sequence similarity based species delimitation approaches will work well for this case.

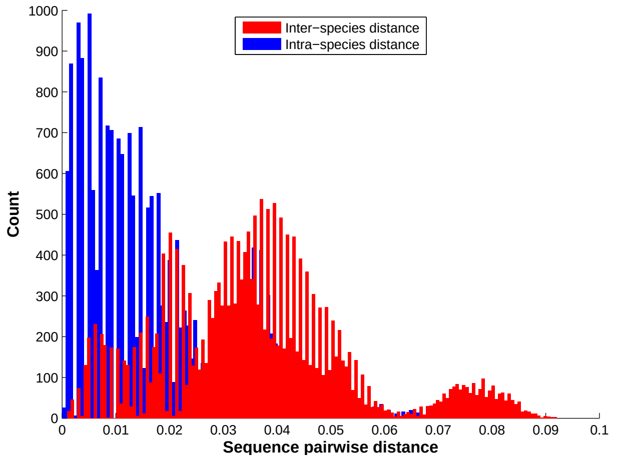

Fig. 3. Histogram of pairwise sequence distances within and among species ($b' = 160$). The two types of pairwise distances overlap, sequence similarity based species delimitation approaches will not work for this case.

Table 3. Species delimitation accuracy on simulated evenly sampled data using the EPA-PTP pipeline

NMI /	b'						Mean
1000bp	5	10	20	40	80	160	(variance)
Full ref.	0.989	0.978	0.962	0.933	0.884	0.836	0.930 (0.003)
90% ref.	0.984	0.972	0.955	0.925	0.876	0.830	0.923 (0.003)
80% ref.	0.976	0.966	0.949	0.921	0.872	0.823	0.917 (0.003)
70% ref.	0.971	0.959	0.943	0.912	0.868	0.816	0.911 (0.003)
60% ref.	0.966	0.956	0.939	0.908	0.860	0.805	0.905 (0.003)
50% ref.	0.962	0.950	0.934	0.904	0.853	0.787	0.898 (0.004)
500bp	5	10	20	40	80	160	
Full ref.	0.986	0.973	0.956	0.927	0.873	0.822	0.922 (0.004)
90% ref.	0.976	0.962	0.947	0.918	0.865	0.812	0.913 (0.004)
80% ref.	0.967	0.954	0.935	0.908	0.858	0.805	0.904 (0.003)
70% ref.	0.957	0.942	0.925	0.896	0.843	0.784	0.891 (0.004)
60% ref.	0.951	0.935	0.916	0.881	0.829	0.780	0.882 (0.004)
50% ref.	0.941	0.928	0.900	0.865	0.812	0.752	0.866 (0.005)
250bp	5	10	20	40	80	160	
Full ref.	0.978	0.968	0.949	0.918	0.863	0.811	0.914 (0.004)
90% ref.	0.967	0.955	0.935	0.907	0.854	0.800	0.903 (0.004)
80% ref.	0.956	0.944	0.926	0.895	0.846	0.786	0.892 (0.004)
70% ref.	0.942	0.926	0.912	0.880	0.830	0.773	0.877 (0.004)
60% ref.	0.927	0.911	0.893	0.861	0.813	0.755	0.860 (0.004)
50% ref.	0.909	0.891	0.871	0.838	0.784	0.732	0.837 (0.004)

Table 4. Species delimitation accuracy on simulated evenly sampled data using the EPA-CROP pipeline

NMI /	b'						Mean
1000bp	5	10	20	40	80	160	(variance)
Full ref.	0.986	0.971	0.950	0.907	0.839	0.759	0.902 (0.007)
90% ref.	0.974	0.959	0.940	0.896	0.831	0.750	0.891 (0.007)
80% ref.	0.963	0.949	0.929	0.890	0.825	0.735	0.881 (0.007)
70% ref.	0.951	0.938	0.916	0.870	0.811	0.728	0.869 (0.007)
60% ref.	0.947	0.929	0.904	0.859	0.791	0.712	0.857 (0.008)
50% ref.	0.941	0.917	0.887	0.839	0.770	0.694	0.841 (0.008)
500bp	5	10	20	40	80	160	
Full ref.	0.978	0.957	0.924	0.874	0.777	0.686	0.866 (0.012)
90% ref.	0.968	0.948	0.916	0.856	0.770	0.681	0.856 (0.012)
80% ref.	0.955	0.932	0.903	0.854	0.764	0.670	0.846 (0.012)
70% ref.	0.942	0.923	0.894	0.835	0.749	0.648	0.831 (0.013)
60% ref.	0.933	0.909	0.873	0.820	0.733	0.649	0.819 (0.012)
50% ref.	0.918	0.899	0.856	0.799	0.721	0.628	0.803 (0.012)
250bp	5	10	20	40	80	160	
Full ref.	0.957	0.934	0.877	0.798	0.683	0.564	0.802 (0.023)
90% ref.	0.945	0.923	0.872	0.788	0.674	0.565	0.794 (0.022)
80% ref.	0.934	0.904	0.859	0.784	0.660	0.554	0.782 (0.022)
70% ref.	0.921	0.901	0.839	0.768	0.653	0.563	0.774 (0.020)
60% ref.	0.907	0.876	0.834	0.758	0.647	0.543	0.760 (0.020)
50% ref.	0.886	0.869	0.812	0.735	0.643	0.549	0.749 (0.017)

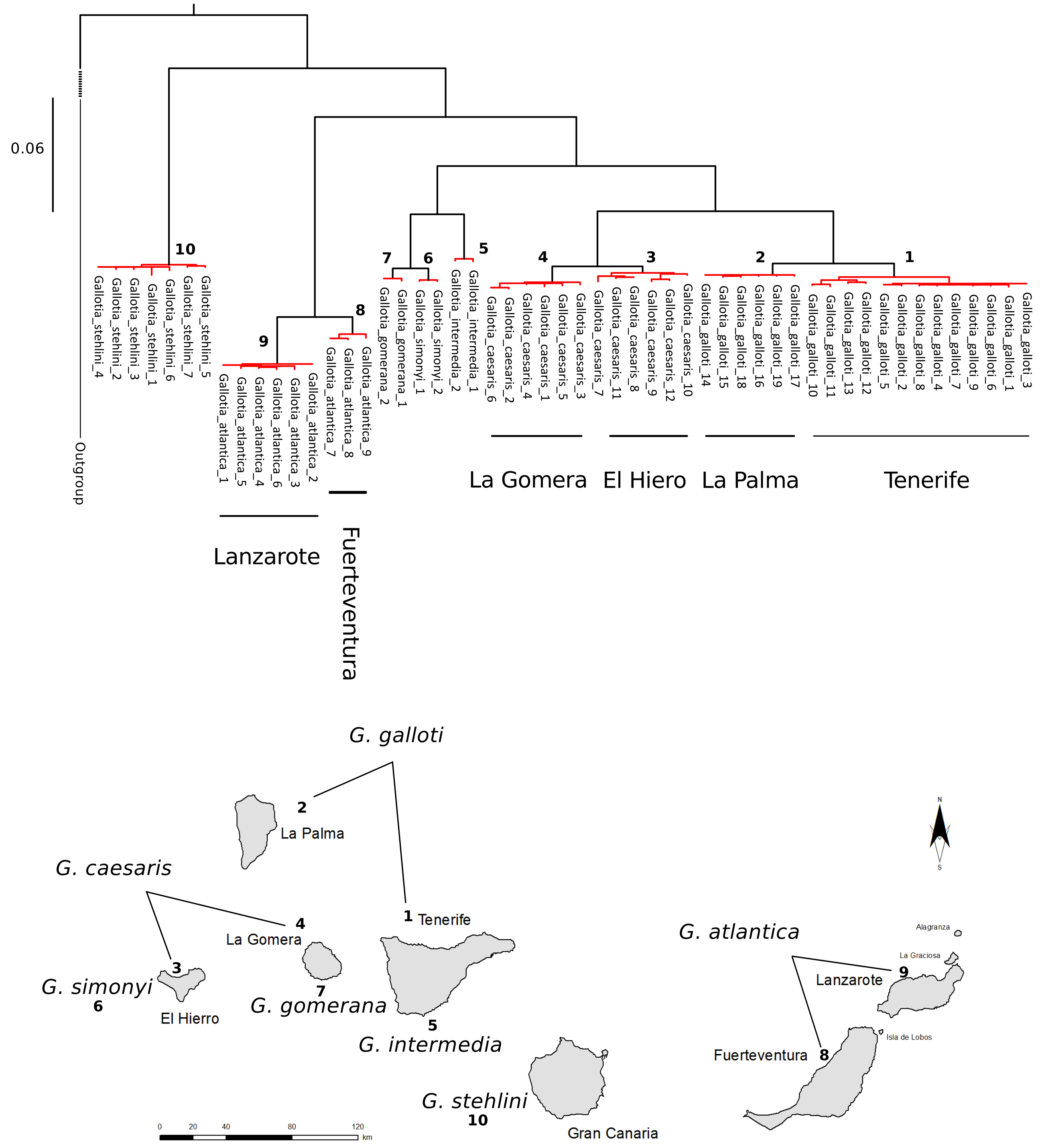

Fig. 4. Phylogenetic relationships within the genus Gallotia as inferred by RAxML. The putative species (1-10) delimited by both GMYC and PTP are highlighted in red. On the map, both the distribution of the Gallotia morphological species and of the species delimited by the two models (1-10) are shown.

Table 5. Species delimitation accuracy on simulated unevenly sampled data using the EPA-PTP pipeline

NMI / 1000bp	b' 5	10	20	40	80	160	Mean (variance)
Full ref.	0.962	0.948	0.923	0.893	0.836	0.791	0.892 (0.004)
90% ref.	0.958	0.945	0.920	0.889	0.835	0.789	0.889 (0.004)
80% ref.	0.951	0.940	0.917	0.884	0.830	0.778	0.883 (0.004)
70% ref.	0.948	0.935	0.913	0.882	0.829	0.775	0.880 (0.004)
60% ref.	0.940	0.925	0.908	0.880	0.824	0.773	0.875 (0.004)
50% ref.	0.936	0.925	0.899	0.878	0.820	0.762	0.870 (0.004)
500bp	5	10	20	40	80	160	
Full ref.	0.969	0.956	0.931	0.899	0.832	0.776	0.893 (0.005)
90% ref.	0.966	0.953	0.925	0.894	0.829	0.768	0.889 (0.005)
80% ref.	0.957	0.943	0.920	0.891	0.822	0.762	0.882 (0.005)
70% ref.	0.951	0.938	0.918	0.883	0.814	0.750	0.875 (0.006)
60% ref.	0.940	0.930	0.950	0.868	0.815	0.741	0.874 (0.006)
50% ref.	0.934	0.920	0.897	0.856	0.801	0.724	0.855 (0.006)
250bp	5	10	20	40	80	160	
Full ref.	0.968	0.954	0.924	0.890	0.819	0.758	0.885 (0.006)
90% ref.	0.960	0.946	0.917	0.881	0.813	0.750	0.877 (0.006)
80% ref.	0.950	0.935	0.911	0.867	0.805	0.739	0.867 (0.006)
70% ref.	0.942	0.925	0.902	0.861	0.796	0.724	0.858 (0.007)
60% ref.	0.927	0.917	0.888	0.843	0.785	0.706	0.844 (0.007)
50% ref.	0.922	0.890	0.873	0.833	0.765	0.685	0.828 (0.007)

Table 6. Species delimitation accuracy on simulated unevenly sampled data using the EPA-CROP pipeline

NMI / 1000bp	b' 5	10	20	40	80	160	Mean (variance)
Full ref.	0.967	0.953	0.923	0.876	0.796	0.716	0.871 (0.009)
90% ref.	0.966	0.950	0.923	0.874	0.792	0.710	0.869 (0.010)
80% ref.	0.959	0.942	0.915	0.868	0.783	0.705	0.862 (0.009)
70% ref.	0.951	0.937	0.910	0.861	0.779	0.693	0.855 (0.010)
60% ref.	0.948	0.934	0.902	0.850	0.774	0.690	0.849 (0.010)
50% ref.	0.949	0.922	0.891	0.826	0.767	0.679	0.839 (0.010)
500bp	5	10	20	40	80	160	
Full ref.	0.962	0.941	0.899	0.843	0.742	0.651	0.839 (0.014)
90% ref.	0.957	0.937	0.897	0.836	0.732	0.635	0.832 (0.015)
80% ref.	0.950	0.930	0.885	0.826	0.733	0.639	0.827 (0.014)
70% ref.	0.942	0.925	0.884	0.824	0.714	0.619	0.818 (0.016)
60% ref.	0.930	0.917	0.871	0.815	0.713	0.615	0.810 (0.015)
50% ref.	0.919	0.901	0.854	0.781	0.692	0.591	0.789 (0.016)
250bp	5	10	20	40	80	160	
Full ref.	0.945	0.922	0.855	0.770	0.647	0.539	0.779 (0.025)
90% ref.	0.935	0.905	0.850	0.766	0.640	0.537	0.772 (0.024)
80% ref.	0.925	0.897	0.829	0.740	0.631	0.524	0.757 (0.024)
70% ref.	0.914	0.887	0.833	0.746	0.640	0.522	0.757 (0.023)
60% ref.	0.901	0.870	0.809	0.743	0.610	0.532	0.744 (0.021)
50% ref.	0.891	0.859	0.799	0.704	0.610	0.508	0.728 (0.022)

Table 7. *Arthropod* data set: Number of estimated OTUs and species for the complete reference data and tree using CROP.

	CROP stand alone			EPA-CROP		
	No. cluster	drop-out	no-match	No. cluster	drop-out	no-match
>= 1 reads	671	33.6%	45.9%	652	7.5%	22.4%
>= 2 reads	465	37.7%	26.7%	538	11.9%	10.4%
>= 5 reads	349	44.6%	13.2%	442	22.5%	4.1%

Sanger data (the reference data set) has a total of 547 OTUs.
